# Plant glucose transporter structure and function

**DOI:** 10.1007/s00424-020-02449-3

**Published:** 2020-08-26

**Authors:** Dietmar Geiger

**Affiliations:** Institute for Molecular Plant Physiology and Biophysics, Julius-von-Sachs-Institute, Biocenter, University of Wuerzburg, 97082 Wuerzburg, Germany

**Keywords:** STP, Sugar transport protein, Glucose transport, Plant photoassimilate partitioning

## Abstract

The carbohydrate D-glucose is the main source of energy in living organisms. In contrast to animals, as well as most fungi, bacteria, and archaea, plants are capable to synthesize a surplus of sugars characterizing them as autothrophic organisms. Thus, plants are de facto the source of all food on earth, either directly or indirectly via feed to livestock. Glucose is stored as polymeric glucan, in animals as glycogen and in plants as starch. Despite serving a general source for metabolic energy and energy storage, glucose is the main building block for cellulose synthesis and represents the metabolic starting point of carboxylate- and amino acid synthesis. Finally yet importantly, glucose functions as signalling molecule conveying the plant metabolic status for adjustment of growth, development, and survival. Therefore, cell-to-cell and long-distance transport of photoassimilates/sugars throughout the plant body require the fine-tuned activity of sugar transporters facilitating the transport across membranes. The functional plant counterparts of the animal sodium/glucose transporters (SGLTs) are represented by the proton-coupled sugar transport proteins (STPs) of the plant monosaccharide transporter(-like) family (MST). In the framework of this special issue on “Glucose Transporters in Health and Disease,” this review gives an overview of the function and structure of plant STPs in comparison to the respective knowledge obtained with the animal Na^+^-coupled glucose transporters (SGLTs).

## Introduction

Larger body size and an increase in complexity is a major trend in animal and plant evolution. Unicellular and multicellular organisms depend on diffusion to supply gases (oxygen, carbon dioxide) and nutrients as well as to remove toxic compounds. However, diffusion represents a slow process and works only across small distances (< 1 mm; [70]). With the appearance of organisms with a larger body size, the surface area cannot meet the needs of its volume in terms of nutrient and gas supply [34, 92]. To deliver essential substances to and from each cell in the body, the need arose to evolve internal transport systems to provide bulk flow transport.

In both, higher plants and animals, long-distance transport of carbohydrates is realized by a system of specialized tubes. In vertebrates, the vasculature forms a circulatory system of arteries, capillaries, and veins. In the closed circulatory system of vertebrates, blood circulates in the vascular system around and between all tissue layers in the body constantly driven by a pump—the heard. Thereby, the diffusion distance for gas, nutrient, and waste transport to individual cells is reduced. Pressure/flow rate, the composition of the blood, and last but not least, the glucose content are strictly controlled.

## Plants do it differently

In plants, the situation is quite different: long-distance transport is not circulatory. More than 500 million years ago, the first plants conquered the land, and about 470 million years ago, the land plant lineage diverged into bryophytes (including liverworts and mosses) and vascular plants [71, 185]. In early land plants, long-distance transport occurs in cells arranged end-to-end in longitudinal files with a simplified cytoplasm and modified end walls. This design increased the intra- and intercellular conductivities for efficient bulk transport of nutrients, photoassimilates, and information [75, 76, 82, 102, 111, 116, 165]. The degree of specialization of conducting cells/tissues increased from non-vascular plants (bryophytes such as hornworts, mosses, and liverwort) to vascular cryptogams (early tracheophytes such as lycophytes and pterophytes) and finally to seed plants [75, 76, 82, 102, 116, 165]. Higher land plants (vascular plants) evolved vascular bundles with two distinct long-distance transport systems—the xylem and the phloem.

The xylem is a specialized plant tissue carrying water and dissolved minerals from the soil via the root to the shoot. This unidirectional transport pathway represents a continuous duct composed of tracheary elements, fibers, and parenchyma cells. Tracheary elements are dead cells with lignified secondary cell walls [97, 135]. These cells are elongated tubes with exaggerated ends that are interconnected through gaps in the cell wall (pits) with the ends of neibouring cells. Bulk flow of xylem sap is driven by transpiration at the leave surface utilizing the water potential difference between the soil and the atmosphere.

**Fig. 1:**
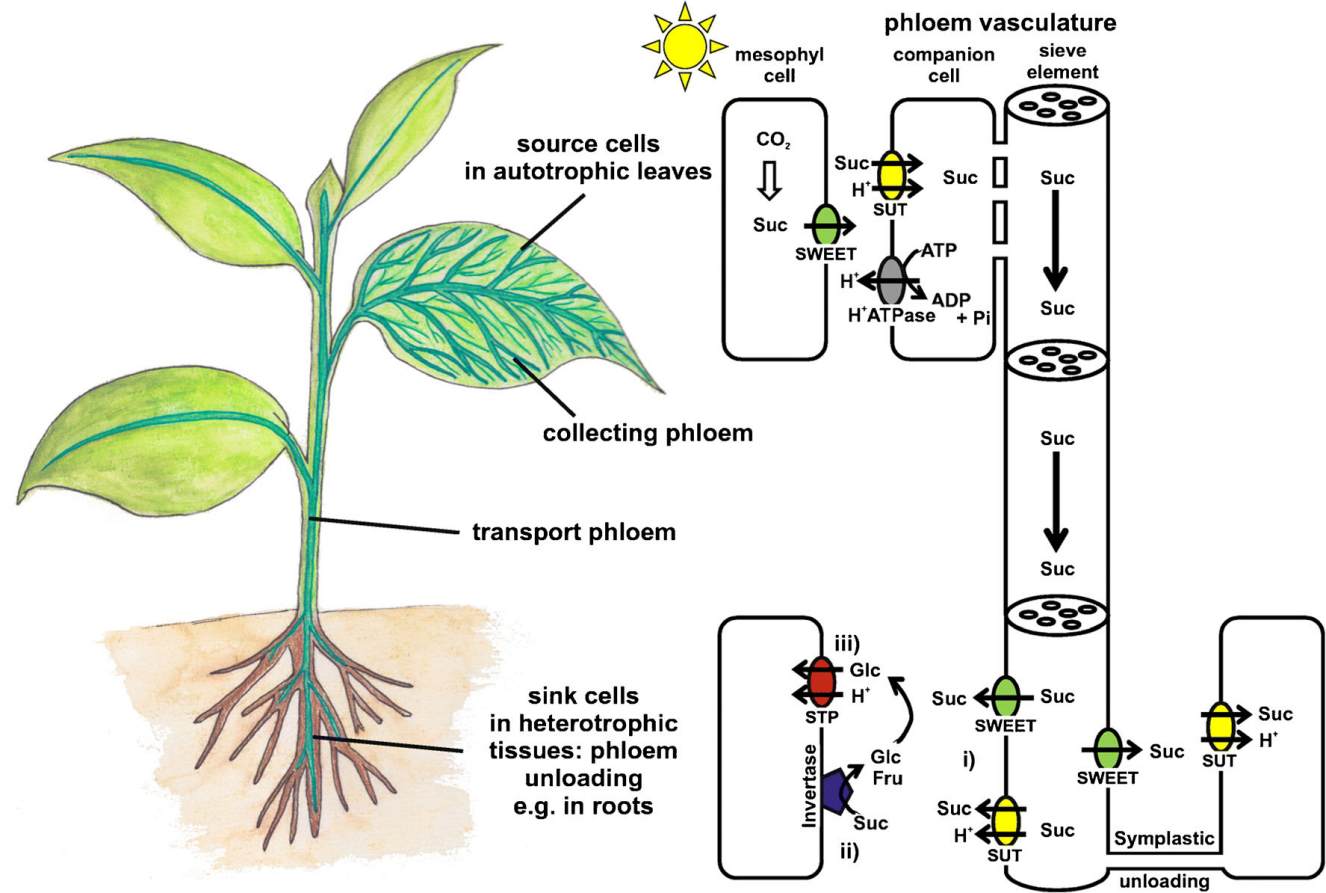
Long-distance transport of sucrose from source to sink tissues in apoplastically loading plants and the involvement of STPs in loading of sink cells with monosaccharides. Left: cartoon of a plant showing the phloem vasculature in dark green. The branched collecting phloem is illustrated only in the right fully developed leaf. Right: Illustration of the loading, the long-distance transport and the unloading of photoassimilates. In plants, photosynthetically synthesized sucrose is released from mesophyll cells to the apoplast (extracellular cell wall space) via SWEET type facilitators. At the source site of the phloem vasculature, H^+^-coupled sucrose transporters (SUTs) accumulate sucrose in the SE/CC (sieve element/companion cell complex—phloem tissue) complex for long-distance distribution throughout the plant body. H^+^-ATPase provide the proton motive force for sucrose loading energized by ATP hydrolysis. To provide heterotrophic sink cells with photoassimilates, sucrose is either imported symplastically via plasmodesmata or via a three-step apoplastic sugar import: (i) sucrose is released from the phloem cells into the apoplasm, (ii) cell wall-bound invertases hydrolyse sucrose to fructose and glucose, (iii) followed by the uptake of the breakdown products into sink cells via STP-type proton-coupled monosaccharide transporters

The phloem transports mainly photoassimilates, amino acids, and information in form of, e.g., phytohormones or small RNAs. The transport in the phloem is bidirectional originating from autotrophic sites where CO_2_ fixation takes place (source tissues) to heterotrophic tissues (sink tissues) such as developing leaves, meristems, roots, and reproductive organs that rely on an adequate sugar-supply by the phloem [55, 141, 165, 169, 170] (Fig. 1). The phloem network is assembled by highly active companion cells (CCs) and adjacent nucleus- and vacuole-free interconnected sieve elements (SEs) with increased intracellular conductivity for efficient bulk transport (Fig. 1). In apoplastically loading plants, the surplus of carbohydrates from source leaves are distributed in the plant throughout the vascular system (phloem) mainly in the form of the disaccharide sucrose (Fig. 1, [186]). Membrane-localized sucrose/H^+^ symporters (SUTs or SUCs) accumulate sucrose in the phloem cells in source tissues where sucrose can reach concentrations of up 1 M [15, 39, 41, 68, 77, 78, 118, 120, 137, 141, 142, 148, 149]. The hydrostatic pressure difference between source (sites of sugar accumulation) and sink (sites of sugar release) tissues drives the mass flow of water and nutrients in the phloem vessels [61, 96].

## Why plants use sucrose instead of glucose for long-distance transport

As stated previously, long-distance transport of photoassimilates is mainly realized in the form of the disaccharide sucrose or less frequently, galactosides of sucrose (sugars of the raffinose family) [118, 186] and not in form of glucose as it is the case in animals. Sucrose is synthesized in photosynthetically active cells from fructose and glucose and is then transported via the phloem to heterotrophic parts of the plant. Sucrose transport is favorable for the following reasons: (i) 1 mol sucrose contains more energy than 1 mol of a monosaccharide such as glucose or fructose, thus using the disaccharide is more energy efficient for transport and storage. (ii) In contrast to fructose and glucose, sucrose represents a non-reducing sugar, meaning that it cannot be oxidized and thus no intermediate reactions with other molecules occur. High concentrations of reducing sugars and amino acid residues can undergo non-enzymatic glycosylation [85] leading to the impairment/damage of critical proteins [7, 159, 181]. Thus, plant cells sequester reducing sugars into the vacuole (Fig. 3) to avoid non-controllable glycosylation of cytosolic proteins [10]. During long-distance transport in living companion cells and sieve tubes of the phloem, sugar concentrations reach several hundred millimolar up to 1 M. The use of hexoses for transport at these high concentrations in the cytosol of phloem cells would lead to massive damages due to glycolysation. In vertebrates, long-distance transport is realized in the form of glucose extracellularly circulating in the bloodstream. The blood glucose concentration is tightly regulated to values in the low millimolar range (5.5 mmol/L global mean fasting plasma blood glucose level in humans; [25]). Thus, plants prefer the non-reducing sugar sucrose (or galactosides of sucrose) for long-distance transport [186]. In sink tissues, sucrose is converted back to glucose and fructose by an enzyme called (cell wall-bound) invertase (Fig. 1, [89, 121, 139, 167]). Subsequently, these hexoses are taken up by sink cells for consumption or storage. The uptake of hexoses across the plasma membrane is facilitated by a family of proton-coupled sugar transport proteins (STPs)—the counterpart of sodium-coupled glucose transporters of the SGLT-family in vertebrates. The function and the structure of these hexose-transporting proteins is the subject of this review.

## Proton-coupled versus sodium-coupled secondary active transport

Transporters mediate the accumulation of substrates by coupling the substrate transport to a thermodynamically favorable symport or antiport of ions (usually Na^+^ or H^+^). Mitchell already in 1963 hypothesized the utilization of free energy stored in electrochemical ion gradients for substrate translocation [91]. ATP-driven primary active pumps—P-type H^+^-ATPases in the case of plants or Na^+^/K^+^-ATPases in animals—establish these electrochemical gradients across biological membranes [95, 104].

Plants utilize proton gradients for secondary active transport whereas animals prefer sodium gradients [98]. When eukaryotes diverged during evolution, plants—just like bacteria—continued using the abundant H^+^ ion to generate proton gradients via P-type H^+^-ATPases at the plasma membrane or vacuole-type ATPases as well as an H^+^-translocating pyrophosphatases at the vacuolar membrane [8, 33, 104, 110, 136, 138]. The activity of P-type proton pumps is important for membrane potential homeostasis and the strong hyperpolarization of plant membranes of more than – 200 mV [47]. Moreover, the accumulation of protons on the outside creates an inward-directed proton gradient with pH values of around 5.5 in the apoplast whereas the cytosolic pH is kept constant at around pH 7.5 [104, 157]. This proton motive force (proton gradients in conjunction with the hyperpolarized membrane potentials) are utilized by H^+^-coupled transporters for substrate transport/accumulation via H^+^/substrate symport or antiport mechanisms. Given a tight coupling between substrate and H^+^ transport, the free energy stored in the electrochemical proton gradient allows for a more than 1,000-fold accumulation of the substrate [20]. Animals evolved the Na^+^/K^+^ATPase for the maintenance of ionic gradients across the plasma membrane [49, 151, 152]. It creates ionic conditions/gradients for critical cellular processes such as secondary active transport of solutes and water, for pH regulation, and for creating and maintaining an electrical potential that is essential for the function of excitable cells in rapid signal transmission systems (e.g., muscles, nerves). Na^+^/K^+^ATPase does not exist in plants.

## Phylogenie and physiology of monosaccharide transporters in plants

**Fig. 2:**
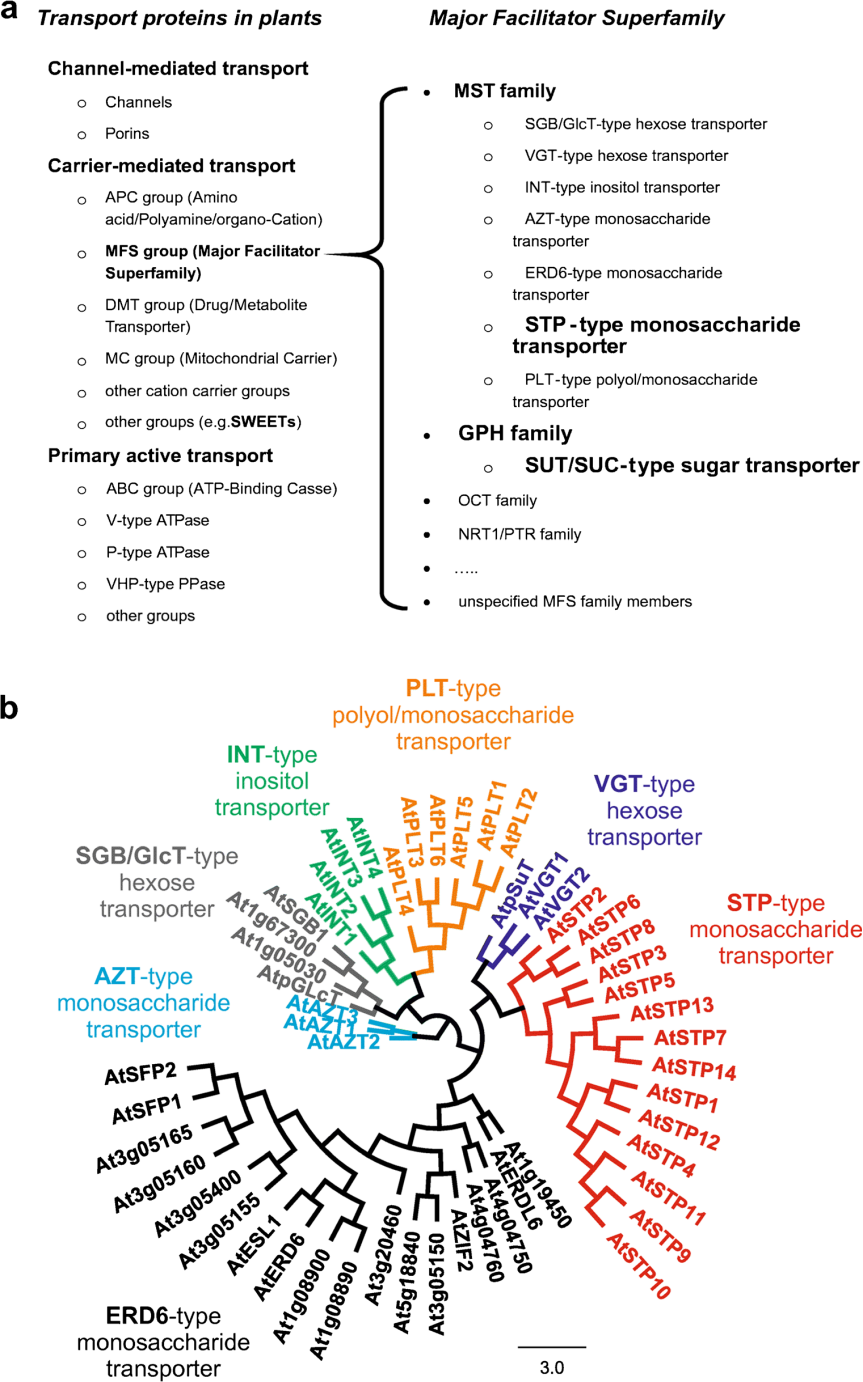
Classification of plant transport proteins. **a** Plant transport processes comprise proteins for channel-mediated transport, carrier-mediated transport, and primary active transport. Secondary active sugar transport proteins are members of the major facilitator superfamily (MFS). The subfamily of monosaccharides transporters (MSTs) is further subdivided in seven groups of transporter families: EDR6 (early response to dehydration)-like, STP (sugar transport proteins), pGlcT/SBG1 (plastidic glucose transporter/Supressor of G protein beta1), INT (inositol or cyclic polyol transporters), PLT (polyol/monosaccharide transporters), AZT/TMT (tonoplastic monosaccharide transporters), and VGT (vacuolar glucose transporters). The family of disaccharide transporters (mainly sucrose transporters) constitute a distinct subfamily within the MFS—the GPH family. Monosaccharide and disaccharide facilitators of the SWEET (sugar will eventually be exported transporters) family are not members of the MFS but group into a distinct structural group of transporters. **b** Phylogenetic tree of the 53 members of MSTs in the model plant *Arabidopsis thaliana*. As mentioned in **a**, the MSTs are subdivided into 7 subfamilies

**Fig. 3:**
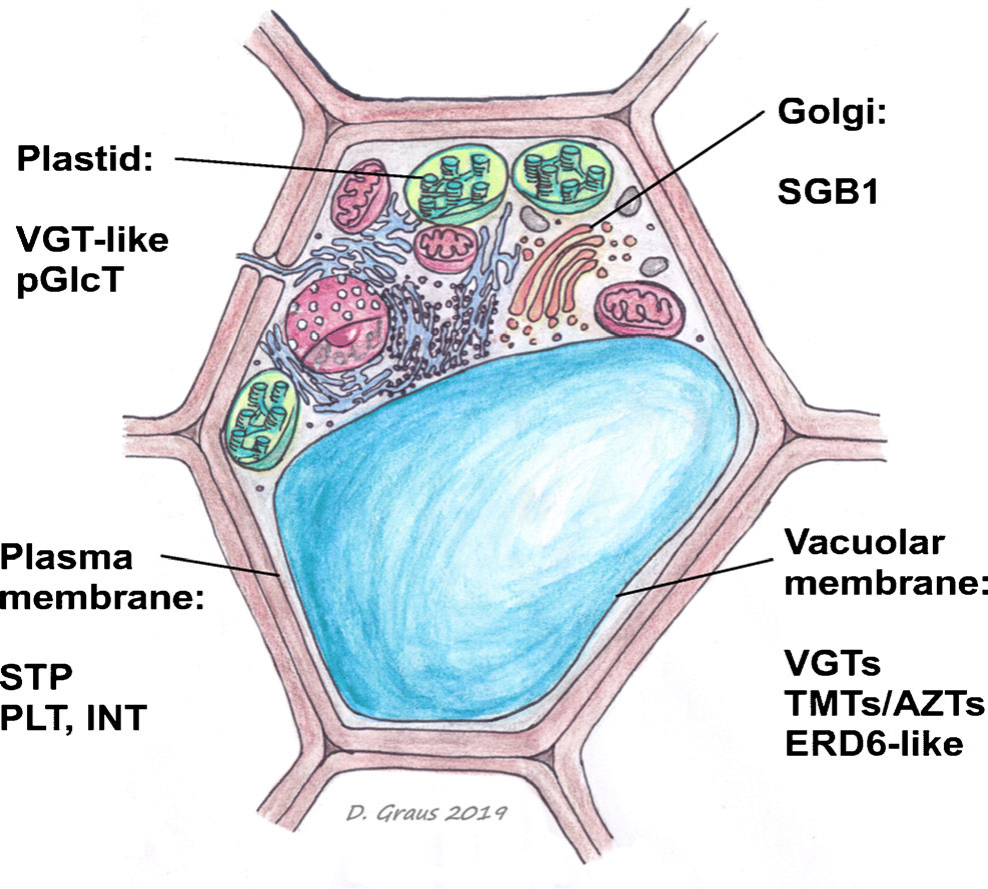
Cartoon illustrating a plant cell. Subcellular localization of MST subfamily groups is indicated. The subfamilies of the plant MST-family consist of EDR6 (early response to dehydration)-like, STP (sugar transport proteins), pGlcT/SBG1 (plastidic glucose transporter/Supressor of G protein beta1), INT (inositol or cyclic polyol transporters), PLT (polyol/monosaccharide transporters), AZT/TMT (tonoplastic monosaccharide transporters), and VGT (vacuolar glucose transporters)

Higher plants express two gene families encoding for monosaccharide transporters: the cation/proton-coupled monosaccharide transporter(-like) superfamily (MSTs, [105, 117]) belonging to the major facilitator superfamily (MFS) and the recently identified SWEET transporter family (sugar will eventually be exported transporters) [23]—facilitators belonging to a group of transporters distinct from the MFS. An overview of the plant transport proteins and their classification into superfamilies/families is presented in Fig. 2a. The phylogenie and evolution of plant MSTs has comprehensively been reviewed by Johnson et al. previously [53, 54]. MSTs are integral membrane proteins that consist of 12 trans-membrane-spanning domains that assemble in a pseudosymmetrical manner to form a central pore/cavity shuttling soluble monosaccharides together with protons across hydrophobic membranes [18, 169]. In the model plant *Arabidopsis thaliana*, 53 members fulfil the criteria to represent a member of the MST-family [105, 117] (Fig. 2b). The Arabidopsis transporters of the MST-family divide into seven subfamilies that are conserved across the seed plants despite the variation in total number of family members in different species [53, 54]. Phylogenetic analysis using expressed sequence tag (EST) data showed that the MST gene family is ancient appearing at least 400 million years ago in land plants and showing differential subfamily expression and lineage-specific subfamily expansions [53]. The seven subfamilies of the plant MST-family (cf. Fig. 2b) consist of two large groups of subfamilies: EDR6 (early response to dehydration)-like and STP (sugar transport proteins) and five small subfamily groups: pGlcT/SBG1 (plastidic glucose transporter/suppressor of G protein beta1), INT (inositol or cyclic polyol transporters), PLT (polyol/monosaccharide transporters), AZT/TMT (tonoplastic monosaccharide transporters), and VGT (vacuolar glucose transporters). An overview of the subcellular localization of MST subfamilies is illustrated in Fig. 3. Although the members of the MST-family cluster into distinct subfamilies, several members exhibit overlapping expression patterns, subcellular localizations, and transport similar sugars. Moreover, many of these transporters transport more than one monosaccharide, though affinities for the substrates vary [17, 127, 140]. Only a few members of the MST(-like) superfamily have been shown to be specific for a single substrate such as AtSTP9 for glucose [131] and AtSTP14 for galactose [113].

Since the STP-subfamily is the best characterized plant subfamily of the MST family and since STPs represent the functional counterparts to human SGLTs, this review focuses on the STP-subfamily. The STP subfamily in the model plant Arabidopsis is encoded by 14 highly homologous STP genes [16] (Fig. 2b). AtSTPs have been functionally studied by heterologous expression in yeast or Xenopus oocytes and were shown to catalyse proton/sugar symport across the plasma membrane into the cell (see Table 1 for more information on functional properties of STPs; [16–18, 100, 123, 127, 131, 132, 134, 161] and references therein).

**Table 1:** Summary of available localization patterns and functional data of the *Arabidopsis thaliana* STPs

Gene name Identifier	Tissue localization	Subcellular localization	Substrate specificity	Substrate affinity	References
AtSTP1 At1g11260	Mainly in leaves and stems	Plasma membrane	Glucose, galactose, mannose, xylose, arabinose	High	[24, 35, 103, 139, 150, 178]
AtSTP2 At1g07340	Early male gametophyte development	Plasma membrane	Glucose, galactose, mannose, xylose, arabinose	High	[154, 161]
AtSTP3 At5g61520	Source leaf	Plasma membrane	Glucose	Low	[19]
AtSTP4 At3g19930	Pollen, root tips, leaf	Plasma membrane	Glucose, galactose, mannose, xylose, arabinose	High	[35, 36, 94, 122, 160]
AtSTP5 At1g34580	Silique and whole seedling	Plasma membrane	Non functional?		
AtSTP6 At3g05960	Fully developed pollen grain	Plasma membrane	Glucose, galactose, mannose, fructose, arabinose	High	[122, 134]
AtSTP7 At4g02050	Multiple tissues with high cell wall turnover except pollen	Plasma membrane	Arabinose, xylose	High	[123]
AtSTP8 At5g26250	Pollen grains, pollen tubes, and ovules	Plasma membrane	Glucose, galactose, mannose, arabinose	High	[122, 123]
AtSTP9 At1g50310	Fully developed pollen grain	Plasma membrane	Glucose, galactose, arabinose	High	[122, 131]
AtSTP10 At3g19940	Germinating pollen and growing pollen	Plasma membrane	Glucose, galactose, mannose,	High	[109, 122, 124]
AtSTP11 At5g23270	Fully mature pollen and growing pollen tubes	Plasma membrane	Glucose, galactose, mannose, xylose, arabinose	High	[122, 132]
AtSTP12 At4g21480	Multiple tissues except pollen	Plasma membrane	Glucose, galactose, mannose, xylose	High	[123]
AtSTP13 At5g26340	Source leaves, vascular tissue of emerging petals, roots, guard cells, cotyledons	Plasma membrane	Glucose, galactose, mannose, xylose, fructose, arabinose	High	[100, 133, 177–179]
AtSTP14 At1g77210	Source and sink tissues, female gametophyte, seed endosperm and in cotyledons	Plasma membrane	Galactose, arabinose	High	[113]

Although gene products of the plant MSTs cluster into phylogenetically distinct subfamilies (Fig. 2b), several transporters show overlapping expression patterns and transport similar sugars. Thus, genetic analyses of many single-gene knockouts of STPs fail to confer a significant phenotype indicating redundancy among similar transporters compensating the loss-of-function of a single transporter. Thus, linking the physiological role of individual STPs determined by heterologous expression to a specific function in plants appeared to be a major challenge. However, by the use of higher order knockouts generated by crossings and CRISPR/Cas9 or the overexpression of sugar transport proteins, major breakthroughs could be obtained [6, 122, 133].

As mentioned previously, photoassimilates are translocated from source to sink tissues over long distances mainly in form of sucrose [186]. Following the breakdown of sucrose to glucose and fructose via cell wall-bound invertases in the apoplastic space (Fig. 1), STPs are believed to transport the monosaccharides into sink cells, such as developing leaves, roots, pollen, flowers, and seeds [3, 17, 73, 87, 123, 128, 140].

**Fig. 4:**
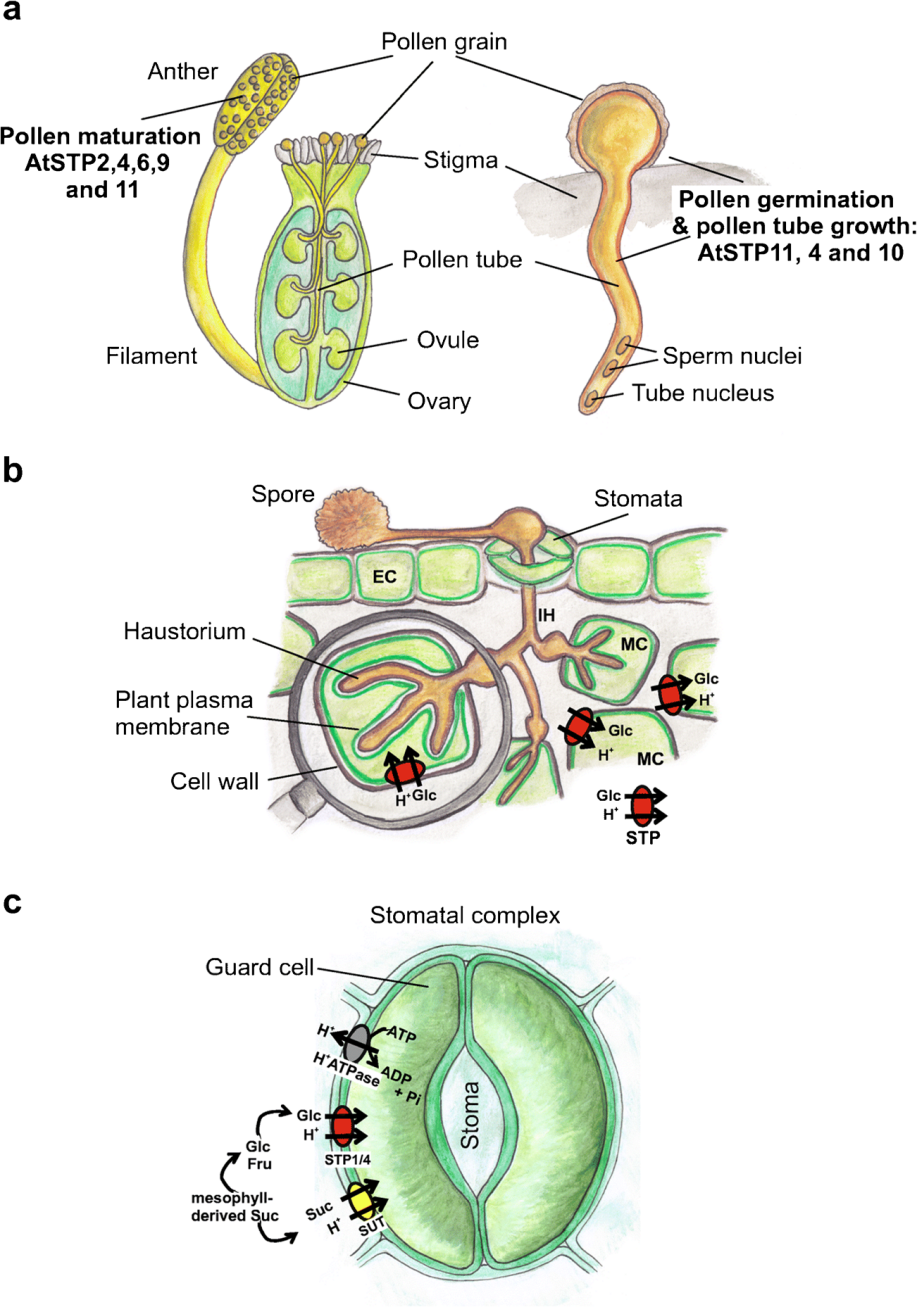
Cartoons illustrating the localization and function of STPs in various plant cell types. **a** In Arabidopsis pollen grain development and maturation in the anther of the flower involve the expression of AtSTP2, 4, 6, 9, and 11. Pollen grains germinate on the stigma. Pollen tubes enter the top of the pistil through the stigma and travel down the style to the ovules, which are contained in the ovary at the base of the pistil. AtSTP4, 10, and 11 are involved in pollen tube germination and growth. On the right, a pollen tube is shown in higher magnification. **b** Cartoon showing a rust infected plant leaf. Germination tubes of spores (S) at the leaf surface enter the leaves through stomata. Primary infection hyphae (IH) propagate through the leaf and penetrate the plant mesophyll cell (MC) wall but not the host plasma membrane to form a feeding structure—the haustorium. During infection, STP1, 4, and 13 expression and activity are induced as part of a defense response [9, 36, 178]. STPs are suggested to lower the apoplastic hexose concentration by moving the sugars into non-infected cells to limit the availability of extracellular saccharides for the pathogen. Another hypothesis implies that the pathogen hijackes STP13 to import sugars into the haustorium or import sugars into the cell that feeds the haustorium with sugars provided from adjacent non-infected cells (Yamada et al. 2016). **c** Guard cells embedded in the leaf epidermis regulate the stomatal opening for gas exchange (CO_2_-uptake, H_2_O release) between the plant and the atmosphere. In Arabidopsis guard cells, STP1 and 4 import mesophyll-derived glucose into guard cells for starch accumulation and light-induced stomatal opening. Thus, mesophyll-derived glucose uptake by guard cells connects photosynthesis with stomatal movements [35]

Besides serving as a nutrient, glucose serves as a signalling molecule that is perceived by the enzyme Hexokinase1 (HXK1) in the cytosol of plant cells [50]. For example, pollen tube germination and growth require metabolic energy that has to be imported from the extracellular space across the plasma membrane in form of carbohydrates (Fig. 4a). Interestingly, however, pollen tube growth arrested upon glucose application [122]. This inhibitory effect was weakened in pollen tubes of a *stp4-6-8-9-10-11* sextuple knockout Arabidopsis mutant as well as in the *hxk1* knockout lines suggesting that STPs are responsible for glucose uptake into the growing pollen tube and that subsequently HXK1 detects the signalling molecule.

Besides supplying sink cells/tissues with sugars, the removal of sugar from the extracellular space has recently been shown to constitute a defense strategy against microbial infection by, e.g., powdery mildew and rust [9, 30, 74, 90, 93, 156, 179] (Fig. 4b). During infection, STP1, 4 and 13 expression, and activity are induced as part of the defense response [36, 179]. Thus, extracellular sugar depletion by STPs restricts the growth and development of microbial invaders (Fig. 4b). Another model suggests that biotrophic pathogens, such as fungi, may hijacke the plant hexose transporter STP13 to import sugars into the haustorium or import sugars into the cell that feeds the haustorium thereby increasing the availability of sugars in the infected cell [9, 179] (Fig. 4b).

Stomatal complexes—composed of two guard cells in the leaf epidermis—regulate the gas exchange (CO_2_-uptake, H_2_O release) between the plant and the atmosphere in response to various stimuli such as, e.g., light, drought, CO_2_ availability, and pathogens. In Arabidopsis, guard cells express the monosaccharide transporters STP1 and 4 (Fig. 4c). Flütsch et al could very recently show that STP1 and 4 are essential for light-induced stomatal opening and starch accumulation in guard cell. These transporters import mesophyll-derived glucose into guard cells connecting photosynthesis with stomatal movements [35].

Glucose transport in humans is accomplished by transporters encoded from two gene families. They are responsible for the absorption of glucose across the small intestine, the reabsorption of glucose from the glomerular filtrate, brain uptake across the blood–brain barrier, and the entire uptake and release of glucose from all kind of cells in the body. One family of glucose transporters facilitate the downhill, passive transport of glucose across cell membranes, the GLUT or SLC2 gene family [164]. Sodium-coupled uphill transport of glucose is mediated by a second gene family, the SGLT or SLC5 gene family [176].

## Transport characteristics of plant monosaccharide transporters of the STP family

STP subfamily orthologs can already be found in the unicellular green algae *Chlorella kessleri* > 450 MYBP ago before Bryophytes evolved [22]. When *Chlorella kessleri* is grown under heterotrophic conditions, glucose serves as sole carbon source. Shifting the algae from carbon autotrophy to heterotrophy, hexose uptake increases more than 200-fold [44, 158]. The transport system for sugars has been associated with proteins encoded by the STP-like HUP (Hexose UPtake) genes (HUP1, 2, and 3). HUP transporters are members of the MFS-family of transporters just like the bacterial model transporter LacY—a H^+^-coupled lactose permease from *Escherichia coli* [56]. Although the helix packing seems identical to the one derived for the *E. coli* lac permease, the mechanism for transport and proton coupling seems to differ between the lac permease and the Chlorella symporter [22]. Detailed functional studies characterized HUP-transporters as H^+^-hexose symporters [62, 64–66], transporting sugars and protons with a stoichiometry of 1:1. Thereby, HUP1 accumulates substrates more than 1000-fold [63]. This accumulation has been explained by the tight coupling between H^+^ and sugar transport, distinct K_m_ values for sugar uptake and release, and partly by differences of velocity constants contributing to uptake compared with those contributing to substrate release [63, 64]. These biochemical and biophysical studies were performed following functional HUP1 expression in different heterologous expression systems, such as yeast strains, *Escherichia coli, Volvox carteri*, and *Xenopus* oocytes [4, 126, 127].

Following the functional description of HUP genes from Chlorella, the hexose transporter STP1 from Arabidopsis represented the first cloned and characterized transporter of a higher plant [127]. Heterologous expression in the fission yeast *Schizosaccharomyces pombe* revealed that AtSTP1 transports glucose with high affinity (20 μM), but is also capable of transporting galactose, mannose, and xylose, however, at lower rates compared to glucose [17, 127]. Interestingly, only AtSTP6 and AtSTP13 transport fructose at significant rates [100, 134] while AtSTP9 is glucose specific [131]. For substrate specificities and affinities of further functionally characterized STP transporters, see Table 1, e.g., [16–18, 123, 124, 132, 161] and references therein). Only little is known about the mechanistic transport cycles of plant monosaccharide transporters. Concerning the kinetic mechanism of monosaccharide-H^+^ symport via STP-like transporters, AtSTP1 is the only member that has been studied in detail so far [11, 12]. Since kinetic parameters of membrane proteins often show pronounced voltage dependence, Borrer et al. (1994, [12]) expressed AtSTP1 in Xenopus oocytes. In contrast to yeast radiotracer flux analysis, heterologous expression in oocytes followed by two-electrode voltage clamp (TEVC) experiments provides the possibility to study kinetic parameter as a function of sugar and H^+^ concentrations in the membrane of a single cell under well-defined membrane potentials. AtSTP1 expressing oocytes were characterized by transport-associated glucose/3OMG (the non-metabolisable 3-O-methylglucose)-induced inward currents [11]. These transport-associated inward currents resemble the symport of protons (positively charged) with the uncharged sugar molecule. Thereby, K_0.5_-values of around 100 μM at pH 6 were calculated. This value in the low micromolar range is well in line with the affinity of AtSTP1 towards glucose determined in yeast (60 μM pH 6; [127]) and hexose uptake assays performed in higher plant protoplasts, cells, or tissue slices with K_m_-values in the range of 100–300 μM [115]. The high-affinity sugar uptake by STPs was later on further confirmed by sugar uptake studies in Arabidopsis seedlings comparing wild-type and an *atstp1*-KO mutant [139]. These data demonstrate that kinetic studies in heterologous expression systems can reflect the properties of sugar transporters in plants.

Furthermore, Borrer et al (1994, [12]) determined a Hill coefficient (n; cooperativity coefficient) for both protons and glucose of 1. n for glucose appeared independent from the external pH, suggesting that AtSTP1 transports H^+^ and glucose in a 1 to 1 stoichiometry. In order to further explore the molecular mechanism of AtSTP1 proton-coupled glucose transport and the underlying transport cycle, pre-steady-state currents (I_pre_) of AtSTP1-expressing oocytes were measured (cf. [13, 21]). Most cation-coupled transporters characteristically display at least two main kinds of electrical currents: besides the organic substrate-associated ion translocation (transport-associated H^+^/Na^+^ currents, I_tr_), co-transporters exhibit pre-steady-state currents (I_pre_, arising from charge movements in the electric field of the membrane) [45, 79, 107, 153]. While the pre-steady-state current is best observed in the absence of substrate, it disappears when the latter is present in saturating amounts [13, 81, 84, 101]. AtSTP1 pre-steady state currents displayed a similar behavior. In the absence of glucose and an external pH of 5.5, these transients could be described by an exponential equation and therein the decay rate constant (s) of I_pre_ and the quantity of displaced charges (Q) could be calculated. The observation that these transient currents vanished at saturating substrate concentrations or at neutral external pH values indicate that I_pre_ resemble the binding of protons to a site of AtSTP1 in the electrical field of the membrane or that conformational changes move charged residues that are located in the electrical field. Based on their kinetic studies, Boorer et al 1994 proposed a six-state transport cycle for AtSTP1 [12], where protons and sugars bind sequentially to the outward facing conformation of the transporter. The loaded transporter undergoes a conformational change presenting the binding sites for the sugar and protons to the cytosolic side of the membrane. Following the release of the substrates, the transporter reorientates in the membrane and thereby finalizes its transport cycle.

SGLT1, the functional animal counterpart of plant STPs, was also expressed and extensively studied in Xenopus oocytes using electron microscopic and optical methods as well as radioactive tracer flux analysis and electrophysiological techniques, summarized in [174]. In contrast to proton-coupled STP transporters, the rate and direction of sugar transport via SGLT1 was shown to depend on the magnitude and direction of the sodium electrochemical potential gradients with a fixed stoichiometry of 2 Na^+^ ions to 1 glucose molecule per transport cycle [31, 114, 162]. Interestingly, protons can drive glucose transport of SGLT1 as well—just like glucose transport via STPs—however, the affinity for sugar is about an order of magnitude lower than in the presence of sodium [173]. Vice versa, dependent on the individual transporter, the magnitude of transport currents but not the affinity towards the substrate of plant proton-coupled sucrose transporters seems to be influenced by high external sodium concentrations [183]. In the case of STPs, the influence of sodium on the hexose transport characteristics was not tested.

The K_0.5_ of SGLT1 for glucose and galactose is equal at 0.5 mM whereas SGLT2 seem to be glucose specific with a K_0.5_ of 2 mM. Just like a thermodynamically perfect machine, the transport direction of SGLT1 was fully reversible with a functional asymmetry between its cytosolic and extracellular face in respect to sugar affinity, sugar selectivity, and inhibitor susceptibility (phlorizin) [31, 106, 107, 114] cf. [20]. These studies revealed an essential ordered six-state kinetic model for Na/glucose cotransport by SGLT1. After the binding of two Na^+^ ions on the external surface, glucose binds to the outward-directed SGLT1 transporter. Due to a conformational change, sodium ions together with one glucose molecule are transported across the membrane, where glucose and the ions dissociate into the cytosol. The empty binding sites then re-orientate from the inner to the external surface to complete the transport cycle [107]. Thus, studies of AtSTP1 [12] and SGLT1 [107] in oocytes revealed a similar essential six-state model.

Plant sugar transporters of the MST family group into the same structural major facilitator superfamily (MFS; [125]) as the model transporters LacY, while the animal Na^+^/Glucose cotransporters SGLTs belong to the large sodium-solute symport family (SSF, [5]). Unfortunately, detailed biophysical and biochemical studies with STP-like transporters in respect to structure-function research, transport cycle, and the associated conformational change vanished in the 90th of the last century. In contrast, SGLT1 and LacY were intensively studied using the cysteine scanning mutagenesis approach in combination with functional studies to draw a mechanistic model of cation-coupled substrate transport across membranes (e.g., [2, 29, 37, 57, 79–81, 86, 146, 184]). Thereby, TM helices and/or individual amino acid residues were identified that contribute to sugar binding/selectivity that are accessible for functional fluorescent molecules such as tetramethyl-rhodamine-6-maleimide (TMRM6) or positions that lead to unfunctional SGLT/LacY mutants following the binding of –SH reagents to the introduced cysteine.

Site-directed alkylation and fluorescence-based methods, as well as single-molecule fluorescence resonance energy transfer (smFRET), support a six-state alternating access model, in which a conformational change is necessary for completion of the LacY transport catalysis [51, 86, 146]. In the case of the electrogenic SGLT1, electrophysiological measurements were combined with voltage-clamp fluorometry (VCF) in *Xenopus laevis* oocytes [79–81, 88]. Thereby, site-specific labeling of an introduced Cys residue with environmentally sensitive fluorophores enabled real-time observation of intramolecular movements under various conditions and positions (e.g., Q457 and G507) in the protein. These fluorescence studies of ligand-induced conformational changes in conjunction with pre-steady-state current analysis associated the charge movements (revealed from I_pre_) with conformational changes of the sodium/glucose cotransporter. When SGLT1 was labelled at position 457, both the level and time course of the change in fluorescence (indicating movements of the specific protein domain) closely followed the charge movements that are a hallmark of the voltage-induced changes in state of the protein [79]. With SGLT1 labelled at position 507, the authors uncovered another slow conformational change probably due to a slow electroneutral step in the transport cycle associated with the release of sodium at the internal membrane surface [80]. Based on many studies using combinations of sophisticated electrophysiological and fluorescence-based techniques, an essential six-state reaction scheme with alternating access of the substrate binding sites for SGLT1 was proposed—similar to the situation in LacY [38, 59, 79–81, 107, 146].

Thus, VCF measurements represent a sophisticated technique to dissect the transport cycle of transporter proteins in partial reactions involved, e.g., in sugar binding and translocation. Neundlinger et al. could add further data in respect to glucose/inhibitor binding to SGLT1 in its natural environment using single-molecule force spectroscopy [99]. The authors resolved the forces and dynamics of glucose/inhibitor binding on the single-molecule level indicating that sugar translocation involves several steps with different temperature sensitivities.

Plant STP transporters were not studied in respect to conformational changes using VCF or similar techniques and thus data concerning transport-associated movements of the protein are not available. The only plant sugar transporter studied with VCF is ZmSUT1—a proton-coupled sucrose transporter originating from a distinct subfamily of the major facilitator superfamily (MFS)—the glycoside-pentoside-hexuronide (GPH) cation symporter family [27, 125] (Fig. 2a).

## Structure of cation-coupled transporters from the MFS family

**Fig. 5:**
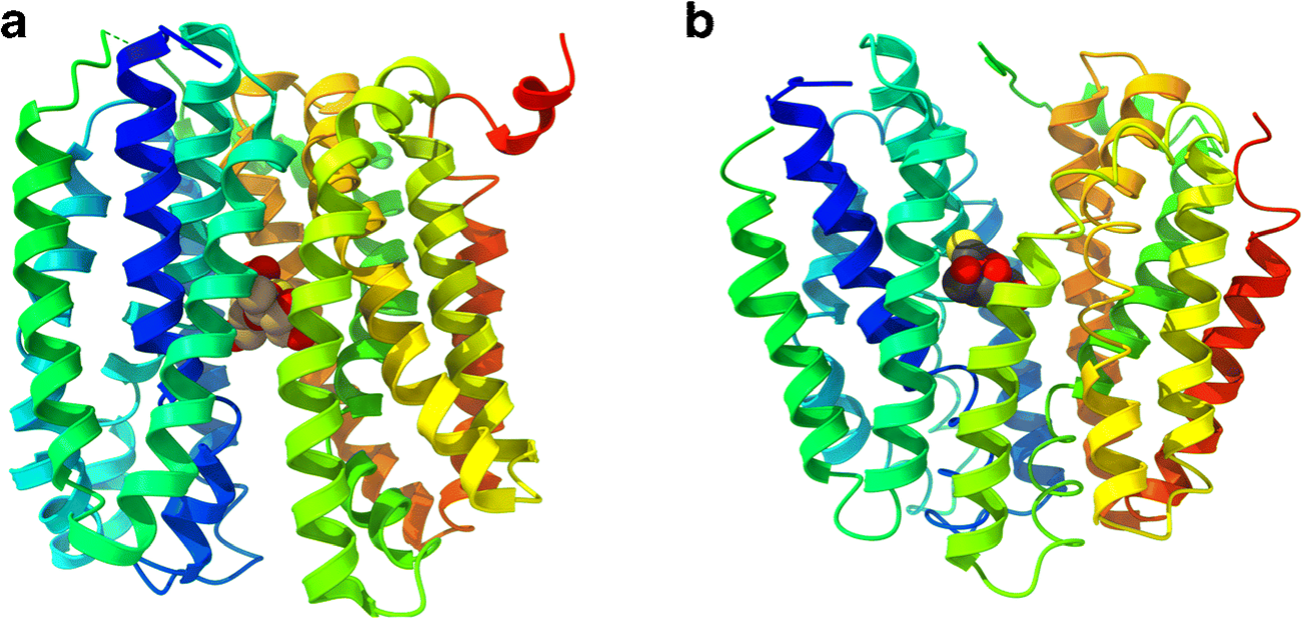
Alternating access model illustrated with the LacY structure. **a** Outward facing (accession number 4OAA) and **b** inward-facing (accession number 2Y5Y) structure of LacY. Structures are downloaded from PDB (www.pdb.org) and presented using UCSF ChimeraX (http://www.rbvi.ucsf.edu/chimerax, version 0.94). The helices were colored using rainbow from N (blue) to C terminus (red). Bound sugars are shown as spheres. The cytoplasmic surface is at the top

Today, the MFS superfamily includes millions of sequenced members distributed ubiquitously across all three kingdoms of life that transport a diverse variety of substrates in either uniport, symport, or antiport ([72, 105, 125, 182]; http://www.tcdb.org/). Until lately, information about the structure of plant sugar transporters was limited to sequence homologies to bacterial and animal cation-coupled transporters. These homologies with non-plant transporters supported the membership of plant hexose transporters in the major facilitator superfamily [105, 125]. In line with members of the MFS, hydropathy analyses with plant monosaccharide transporter sequences predicted 12 transmembrane helices with the N- and C-terminus located in the cytosol for the analyzed proteins (e.g., [119, 129]). Crystal structures of LacY (lactose/H^+^ permease from E. coli) and other MFS transporters elucidated the three-dimensional organization of this type of transporters ([2, 5] and references therein). It could be shown that these transporters consist of two pseudo-symmetrical halves formed by six TMD (transmembrane domains) bundles each, which are connected by an extended cytoplasmic loop. The sugar-binding site at the interface of the two halves is placed in the middle of the membrane. Biochemical and biophysical data resolved irreplaceable and highly conserved amino acid residues involved in substrate binding and proton transduction [58]. In high-resolution X-ray structures of LacY, a large internal cavity is open towards the cytoplasm but closed to the periplasm, representing the inward-facing conformation of the transporter [1, 42, 43]. Using double Gly-to-Trp mutants to introduce bulky side chains on the periplasmic side between the N- and C-terminal sixhelical halves of LacY [145] and later on with the help of nanobodies, the periplasmic-open conformation and transient conformers of LacY could be stabilized and crystalized [52, 69, 143, 144]. Thus, X-ray structures of LacY provide snapshots of local and global conformational changes that allow the sugar- and proton-binding sites gain alternating access to either side of the membrane (Fig. 5). These observations are underpinned by pre-steady-state kinetic analysis and multiple biochemical as well as spectroscopic approaches that—together with the structural data—document the alternating access mechanism underlying the symport of H^+^ and sugar in LacY (Fig. 5).

Based on these extensive studies and the high-resolution crystal structures, the LacY transporter often served as a template for 3D structural models of related transporters to predict tertiary structures and functional domains [40, 46, 108, 112, 166, 172, 187]. Sequence alignments and modelling attempts between LacY and plant sugar transporters showed only low sequence identities and only few of the essential amino acid site chains of LacY are present at identical positions in the respective TMDs of the plant counterparts. Thus, 3D modeling of plant sugar transporters may predict the overall tertiary structure only and will not explain the mechanism of high-affinity substrate transport in, e.g., STPs.

**Fig. 6:**
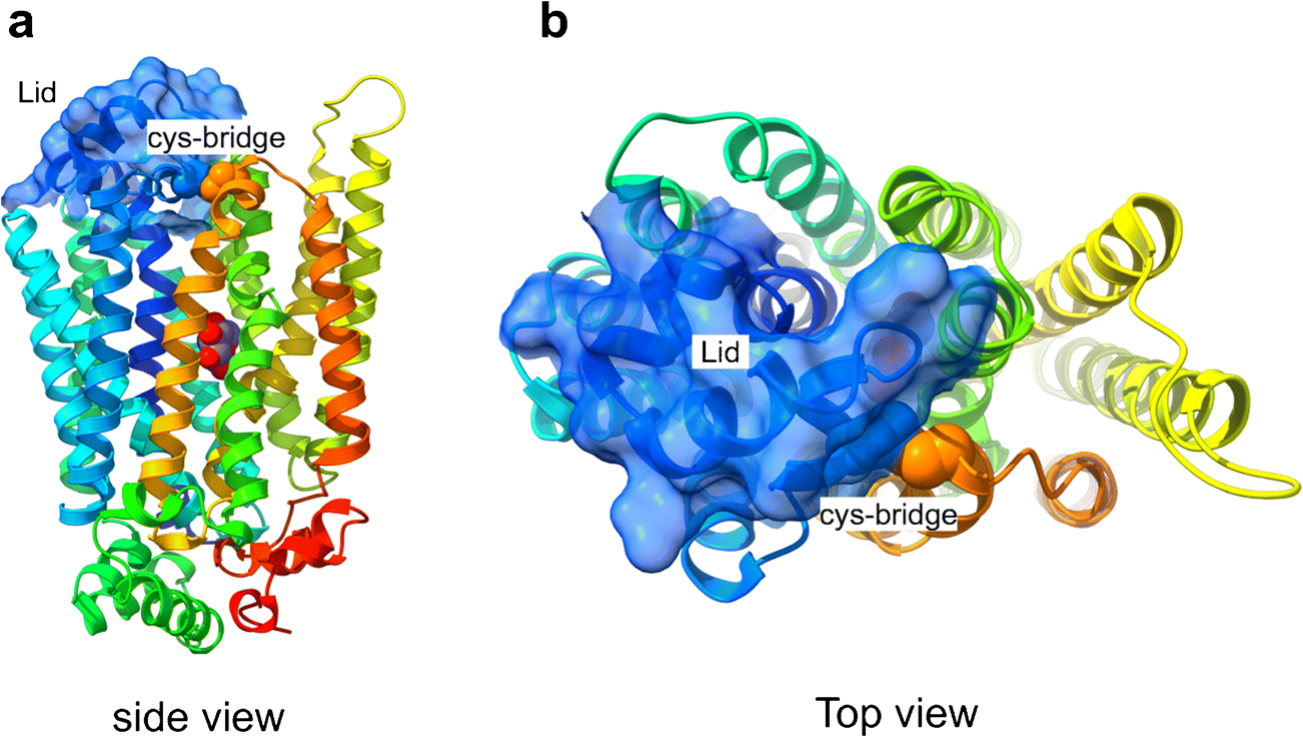
A structural perspective of the *Arabidopsis thaliana* monosaccharide transporter AtSTP10 from side (**a**) and top (**b**) perspective. The structure represents an outward facing occluded state of the sugar transporter in complex with glucose (shown as spheres). Structure (accession number 6H7D) is downloaded from PDB (www.pdb.org) and presented using UCSF ChimeraX (http://www.rbvi.ucsf.edu/chimerax, version 0.94). The helices were colored using rainbow from N (blue) to C terminus (red). The surface of the STP-specific helix-helix-loop-helix motif (Lid) in the loop between TMD1-2 is illustrated in transparent blue. The cysteins involved in the disulfide bridge locking the N- and C-terminal domains together are shown as blue (Cys77) and orange (Cys449) spheres

Very recently, Paulsen and co-workers determined the crystal structure of AtSTP10 in an outward facing occluded state with glucose bound in a central binding site at a resolution of 2.4 Å [109]. Just like AtSTP1, the pollen tube-expressed AtSTP10 represents a typical proton-driven symporter that besides glucose transports galactose and mannose with high affinity (low μM range, Table 1) [124]. The overall fold of AtSTP10 is reminiscent of the typical major facilitator structure with 12 TMDs divided into two pseudosymmetrical halves each consisting of six TM helices and a central sugar binding site located between the N- and C-terminal domains [109] (Fig. 6). Besides the common basic structural organization shared between the members of the MFS, the crystal structure of AtSTP10 revealed an unexpected feature: a “helix-helix-loop-helix” domain in the first extracellular loop between TMD1 and 2 that covers the entry pathway of sugars and was thus named “Lid” domain. This protrusion of the loop between TMD1-2 is covalently linked to the C-terminal domain by a disulfide bridge locking the N- and C-terminal domains together (Fig. 6). Due to the position of the Lid domain, there is no clear entry pathway for the substrate as seen in other major facilitators [26]. Interestingly, the Lid domain resembles a conserved feature found in sequences of all STPs and was thus far not found in any other member of the MFS.

How to explain the high affinity of STP transporters? Based on the crystal structure of AtSTP10, the authors found close interactions of glucose with three residues in the N-terminal domain (Phe39, Ile184, and Leu43) that create a hydrophobic interaction surface for the sugar. This tight and hydrophobic interaction surface cannot be found in low-affinity human GLUTs or bacterial sugar/H^+^ transporters, where the distance is longer and the interacting residues are polar [26, 48, 155]. High-affinity protein-ligand and protein-protein interactions seem to be based on tight hydrophobic interaction interfaces [60, 147] indicating that the high-affinity transport via STPs results from the tight binding of glucose to the identified hydrophobic interaction surface [109].

Below the Lid domain, only two charged residues (Asp42 and Arg142) were found in the TMD region that could act as proton donor/acceptor pair needed for proton translocation and the coupling between cation (H^+^) and sugar transport [14]. The position within the structure is similar to the respective position of other proton-coupled transporters of the MFS [48, 171] and mutants of AtSTP10 at positions Asp42 and Arg142 did not transport glucose anymore [109]. The authors suggest that protonation at Asp42 leads to a displacement of Arg 142 and thus a movement of the outer part of TMD1. This involves the movement of the glucose interaction sites Phe39 and Leu43, which subsequently leads to the tight coordination of the substrate. This scenario provides an explanation for the tight coupling of the proton-motive force and the transport of sugar. Due to the lack of structural information about the inward facing conformation of AtSTP10, the mechanism of sugar/H^+^ release and the accompanying conformational change remain to be elucidated.

Why does STPs possess a Lid domain? Based on their structural and functional findings, Paulsen et al. propose that the Lid domain via the disulfide bridge locks the N- and C-terminal six-helix bundles together (see Fig. 6) and that a conformational change must occur to allow the entry of the substrate to the binding site in the middle of the TMDs. The Lid isolates the proton donor/acceptor pair as well as the sugar-binding site from the extracellular environment and thereby creates a local milieu for efficient transport of glucose at physiological pH values of around pH 5 to 6. Respective mutants that do not possess a disulfide bridge between the Lid domain and the C-terminal domain displayed a pH optimum for transport of glucose that was markedly shifted to more acidic pH values.

In contrast to MFS members, SGLTs group into the large family of solute sodium symporters (SSS) [175, 176] that cotransport Na^+^ with sugars or in the case of SGLT3 act as glucose sensor [28, 130]. The physiological roles and the human diseases related to the disfunction of SGLT family members have been well characterized (detailed summaries of the pathophysiology of the SGLT family is the topic of other reviews within this issue). SGLTs share an alternating-access mechanism with tight coupling between Na^+^ and sugar transport [80, 83].

In 2008/2010, Faham et al. and Watanabe et al., respectively, [32, 168] could resolve the crystal structure of the *Vibrio parahaemolyticus* Na^+^/galactose symporter (vSGLT) with ~ 3.0 Å in the inward-facing occluded and the inward-facing open state. Experimental and in silico studies predicted 14 TMDs with extracellular amino and carboxy termini for SGLT1, which was finally confirmed by the crystal structure of vSGLT [162, 163]. The core structure consists of inverted repeats of 5 TMDs (TM2 to TM6 and TM7 to TM11) and galactose is bound in the center of the N-terminal and C-terminal 5 TMD bundles, occluded from the outside solutions by hydrophobic residues. Thus, despite of the similarity of the overall fold that is shared between vSGLT and LeuT (5+5) fold transporters (cf. [5]), vSGLT has its own specific structural modifications: vSGLT contains 14 TMDs and extracellular amino- and carboxyl-termini [32, 168]. Interestingly, the core structure of vSGLT is similar to the leucine transporter (LeuT) originating from a different gene family [67, 180]. This similarity, the vast amount of biophysical data of studies with SGLTs [99, 175], and the well-characterized structures of LeuT were sufficient to model the outward-facing confirmation of vSGLT and to predict sodium and sugar-binding sites as well as the structural movements accompanying the transport of Na^+^/sugars via SGLTs [32, 168].

Thus, having the structures from the plant AtSTP10 and its bacterial/animal counterpart in hands, it becomes clear that there are similarities but also pronounced differences among the different transporter families. These information could now help to perform detailed structure-function analysis to better understand the high-affinity transport mechanism of plant MSTs of the STP family.

## Future opportunities

Pronounced progress in identifying, characterizing, and determining plant sugar transporters has been made within the last decades. However, in comparison to the deep biophysical, biochemical, and structural characterization of bacterial and animal model transporters, the plant functional orthologs have been studied in less detail so far. The high impact of basic research on bacterial and animal transport proteins, such as LacY, LeuT, SGLT, and GLUT, results at least in part from the incitement to understand the transport mechanisms in molecular/atomic detail to develop medications to cure diseases related to those transporters or transported substances. As sessil organisms, plants synthesize high numbers of various secondary metabolites; thus, plant transport proteins seem to be less sensitive to chemicals (agonists/antagonists/inhibitors) or alternatively, the pharmacology of plant transport proteins was not sufficiently tested so far. The incitement of the agricultural industry, funding agencies, and basic research at universities to understand plant transport proteins on the atomic level and subsequently to manipulate or protect crops from diseases seems to be less developed than the interest in animal/bacterial transporters.

With the first X-ray structure of a plant sugar transporter in hands (AtSTP10; [109]), the door is now open to combine biochemical and biophysical studies to understand the molecular mechanism of monosaccharide transport in plants. Therefore, further structural information about AtSTP10 in different conformations needs to be obtained to gain high-resolution snapshots of individual steps in its reaction cycle. These structural data have to be complemented with dynamic information about how sugar, protons, and the membrane potential induces local or global conformational changes and to understand the tight coupling between the proton motive force to the transport of sugar.

These studies have to include, e.g., site-directed mutagenesis, sophisticated electrophysiology, structural biology, molecular dynamics simulations, and fluorescence-based techniques to dissect the transport cycle of STP10 into individual steps. Thereby, the research on bacterial and animal transporters can guide the studies with plant transporters. The comparison of structural and functional features of different transporters will finally reveal fundamental principles of sugar transport with similarities and distinctions that (co-)evolved driven by specific challenges during the evolution of transport proteins in the three kingdoms of life.

## Data Availability

N/A
